# Graph-BERT and language model-based framework for protein–protein interaction identification

**DOI:** 10.1038/s41598-023-31612-w

**Published:** 2023-04-06

**Authors:** Kanchan Jha, Sourav Karmakar, Sriparna Saha

**Affiliations:** 1 Department of Computer Science and Engineering, Indian Institute of Technology Patna, Patna, Bihar 801103 India; 2 Department of Computer Science and Engineering, National Institute of Technology Durgapur, Durgapur, West Bengal 713209 India

**Keywords:** Biotechnology, Computational biology and bioinformatics

## Abstract

Identification of protein–protein interactions (PPI) is among the critical problems in the domain of bioinformatics. Previous studies have utilized different AI-based models for PPI classification with advances in artificial intelligence (AI) techniques. The input to these models is the features extracted from different sources of protein information, mainly sequence-derived features. In this work, we present an AI-based PPI identification model utilizing a PPI network and protein sequences. The PPI network is represented as a graph where each node is a protein pair, and an edge is defined between two nodes if there exists a common protein between these nodes. Each node in a graph has a feature vector. In this work, we have used the language model to extract feature vectors directly from protein sequences. The feature vectors for protein in pairs are concatenated and used as a node feature vector of a PPI network graph. Finally, we have used the Graph-BERT model to encode the PPI network graph with sequence-based features and learn the hidden representation of the feature vector for each node. The next step involves feeding the learned representations of nodes to the fully connected layer, the output of which is fed into the softmax layer to classify the protein interactions. To assess the efficacy of the proposed PPI model, we have performed experiments on several PPI datasets. The experimental results demonstrate that the proposed approach surpasses the existing PPI works and designed baselines in classifying PPI.

## Introduction

Proteins are essential to all living species as they are involved in every cellular process and function. All proteins are made up of twenty standard amino acids (AAs), considered the building blocks of proteins, which are arranged differently for different proteins. How these amino acids are arranged for each protein decides each protein’s function. Mostly, proteins need a partner to work with, such as proteins, DNA, or RNA. The functionality of a protein in a cell is constrained if it exists alone, but when all the necessary proteins are present, they cooperate to perform their functions. protein–protein interactions (PPI) happen when two or more proteins have physical contact due to some biochemical events or communicate through a signaling process^1^. Through these interactions, proteins control and assist various cellular functions, and biological processes, including cell signaling, cellular transport, muscle contraction, catalytic activity, DNA transcription, and replication^2,3^.

The thorough study of PPI has aided in modeling functional pathways to demonstrate the molecular mechanisms of cellular processes and in discovering drug targets^4^. As PPI involves more heterogeneous processes, it is necessary to identify and analyze the consequences of those interactions to gain a deeper understanding of their significance in the cell. Collecting a good amount of PPIs data for various species has become more accessible due to large-scale experimental PPIs detection technologies like yeast two-hybrid (Y2H) screens, tandem affinity purification (TAP), mass spectrometric protein complex identification (MS-PCI), and other high-throughput biological techniques. Despite significant efforts to uncover PPIs, there is still a considerable gap between PPIs identified through experiments and PPIs found in nature. Experimentally identified PPIs cover only a small percentage of PPI networks. The rationale is that the entire PPI network cannot be explored using these experimental methods for PPI detection due to their costs and time requirements. Additionally, because the output is affected by the experimental setting and device resolution, there is a high rate of false positives and negatives in the PPI data obtained using these methods^5^. Therefore, high-throughput computational approaches are essential for identifying protein interactions. These computational-based techniques, when used in conjunction with experimental methods, also enhance the quality and accuracy of PPI prediction^6^.

Intelligent computational approaches based on ML/DL are urgently needed to automate the identification and analysis of the interactions between proteins. Many computational techniques have been introduced to investigate PPI networks in organisms, using different protein information types, such as protein sequence, structure, gene co-expression, gene ontology, etc. Sequence-based features are widely utilized to identify the interactions between proteins among these sources of protein information due to their ease of availability^7^. The sequence-based features can be categorized into two categories: manually crafted features and auto-engineered features. The former method requires background biological knowledge to convert the symbolic representation of protein sequences into real-number feature vectors, whereas the latter method does not need any background knowledge. Most of the previous studies for PPI prediction have used manually crafted features as the initial feature vector, which are fed into the deep learning models to capture relevant features from the raw features. Some researchers have recently devised techniques that provide fixed-length embeddings (*per-protein and per-residue)* for variable-length protein sequences^8^. The idea is to view each amino acid protein sequence as a sentence and each amino acid as a character or word. The language models from the NLP domain are borrowed and generate the embeddings for sequences with some modifications to handle the large length of amino acid sequences.

A recent trend has seen graph neural networks picking up pace and becoming an essential tool in graph-based applications. For example, Huang et al.^9^ have used graph convolution to identify the associations between miRNA and drug resistance. The problem is constructed as a link prediction problem to predict their associations. Graph neural network-based approaches have been proposed to address other problems, such as chemical stability prediction^10^, protein interface prediction^11^, protein interaction prediction^12^, protein solubility prediction^13^, and to model the adverse effects of polypharmacy^14^. Yang et al.^15^ have proposed a model for PPI prediction utilizing PPI network topology as a graph and the conjoint-triad (CT) method to get the node’s features. In a PPI network graph, each node represents a protein, and an edge defines the relationship between protein pairs (interacting or non-interacting). Authors have used a signed variational graph auto-encoder (S-VGAE), which employs graph convolution layers, to learn the hidden or compact representations of nodes. The learned representation of proteins in pairs is concatenated and fed into the neural network classifier to predict PPI.

All the above-mentioned studies solving different biomedical problems have used graph convolutional networks (GCN)^16^. The existing graph neural network variants, including graph convolutional networks, may suffer from the problems of suspended animation^17^ and over-smoothing^18^ because of the overreliance on the graph links. To address these issues, the Graph-BERT model^19^ based on attention mechanisms^20,21^ has been proposed, in which training is done by sampling nodes with their context, known as linkless subgraphs, from the input graph. In this work, we present a framework that employs the Graph-BERT and *SeqVec* language model^22^ to predict PPI more effectively.

The contributions of our proposed work are listed below:

- We devise the PPI prediction problem as a node classification problem by building a graph where each node represents a protein pair and an edge defines the relationship between two pairs if there is a common protein between them.
- We develop a framework utilizing the Graph-BERT model to learn the hidden representations of graph nodes by focusing on linkless subgraphs instead of a complete input graph and *SeqVec* language model to generate embedding for each node in a graph.
- We demonstrate that the proposed graph-based approach to predicting PPI is better than the existing graph-based approach^15^.

## Related works

So far, several computational approaches have been put forth to categorize the interactions between proteins. Sarkar and Saha^23^ have reviewed the computational methods employing machine learning algorithms such as SVM, naive bayes, decision tree, and random forest, along with the different sources of protein information that are input to these algorithms. SVM is the most widely used machine learning algorithm to predict PPI among these algorithms^24–27^. Later, some studies have shown that the random/rotation forest-based approaches perform better than the popular SVM-based PPI methods^28,29^.

With technological advances, researchers have started using deep learning algorithms to predict PPI and achieved better results than conventional machine learning-based approaches. For example, Wang et al.^30^, and Sun et al.^31^ have employed a stacked auto-encoder to learn the hidden or compact representation of input features, which are derived from protein sequences. Patel et al.^32^, and Zhang et al.^33^ have devised the protein interaction prediction tool named as DeepInteract and EnsDNN, respectively, both employing the deep neural network (DNN). In order to extract information from the protein sequence, Wang et al.^34^ have proposed a sequence-statistics-content (SSC) protein sequence encoding format. A 2D convolutional neural network is then used to predict the PPI utilizing SSC. All the studies discussed above have used hand-engineered or manually crafted features as inputs to the classifiers. The rapidly developing deep learning technique that enables automatic feature engineering is having immense success in numerous fields. Some studies on PPI have also used auto-engineered features. For instance, Li et al.^35^ have presented a framework utilizing a deep neural network, DNN-PPI, which considers features learned from protein sequences automatically to predict PPI. In this framework, the sequences of two interacting proteins are input to the two separate sequential layers of encoding, embedding, convolutional neural networks (CNN), and long-short-term memory (LSTM). The two outputs from the LSTM layer are then merged and fed into a dense layer to predict labels. Gonzalez-Lopez et al.^36^ have suggested using an embedding technique with a recurrent neural network-based architecture to predict interactions directly from protein sequences. Chen et al.^37^ have proposed a deep learning-based framework, PIPR, composed of convolutional layers with pooling followed by bi-directional residual gated recurrent units. These two components are stacked alternatively to extract local and global features. The input to the PIPR model is the pre-trained embeddings of protein sequences.

The deep learning algorithms can handle high-dimensional data and capture hidden associations in data with multi-modal distributions. Recently some studies on PPI^38,39^ have utilized multiple sources of protein information such as protein sequence, 3D structure, and gene ontology. They have developed deep multi-modal PPI models to predict the protein interactions utilizing different combinations of the available protein information. They have used the latest deep learning algorithms to extract relevant features from these modalities. A living organism’s PPI data *(interacting and non-interacting)* can also be defined graphically as a PPI network, where nodes represent proteins and interactions between them are represented by edges. A graph-based deep learning model has been developed by Yang et al.^15^ to predict protein interactions from PPI network topology. The authors have used a signed variational graph auto-encoder to learn low-dimensional features from graph structure. The conjoint-triad (CT) method, which belongs to the manually crafted sequence-based method, has been utilized to get a node’s feature vector.

Current work is also a step toward utilizing graph-based neural networks to predict the interaction between the proteins. We formulate the PPI prediction as a node classification problem, where each node represents a protein pair (interacting or non-interacting). We have used the Graph-BERT model^19^ to learn the hidden representation of the node’s feature vector obtained by the language model *SeqVec*^22^ directly from protein sequences.

## Materials and methodology

This section deals with the datasets that are used to substantiate the proposed approach, followed by the formulation of PPI prediction as a node classification problem. The proposed approach to predict protein interactions comprises three modules: protein sequence embedding; learning hidden representations of graph nodes using a graph transformer model; and PPI classification using learned representations. This section discusses each module in detail.

### Datasets

The Pan’s human PPI dataset (http://www.csbio.sjtu.edu.cn/bioinf/LR_PPI/Data.htm)^40^containing both positive and negative samples, has been used as a benchmark dataset to validate our proposed approach. The HPRD (https://www.hprd.org/) dataset serves as the source for the interacting pairs. The non-interacting dataset is comprised of non-interacting pairs from the negatome database^41^ as well as pairings of proteins from different subcellular localizations. The Swiss-Prot database has information related to the proteins’ subcellular localization. In this dataset, protein sequences with less than 50 amino acids and those with unknown amino acids were excluded. The self-interacting protein pairs were also discarded from this PPI dataset.

We have also used the datasets of other species, such as *E. coli*, *Drosophila*, and *C. elegan*, provided by Guo et al.^42^ to evaluate the efficacy of the suggested approach. To create non-redundant PPI subsets of these species, the CD-HIT program^43^ has been used. A protein is discarded if its sequence identity is high ($>40\%$) or if it has fewer than 50 amino acids. The characteristics of these datasets are tabulated in Table 1.

**Table 1:** Statistics of PPIs datasets.

Dataset	# Positive samples	# Negative samples
Human	36,545	36,323
*E. coli*	5576	4031
*Drosophila*	19,712	14,900
*C. elegan*	2877	1670

### Problem definition

Given the PPI database and protein sequences, we build the graph $G_{ppi} = (V,E)$, where $V = \{P_{12}, P_{13}, P_{23}...\}$ is the set of protein pairs *(interacting and non-interacting)* or nodes, and *E* is the set of edges defined between two nodes if there is a common protein between them. The set of initial feature vectors generated from protein sequences for all nodes $v \in V$ in a graph $G_{ppi}$ built from the PPI network is represented by *X*. The objective is to predict the labels for all nodes (protein pairs) in a graph using the learned low-dimensional representations of nodes, which are passed through the fully connected (FC) layer followed by an output layer (sofmax), expressed by Eq. (1):

1 $$\begin{aligned} Y = softmax(FC(Graph-BERT(X, E))) \end{aligned}$$

**Figure 1:**
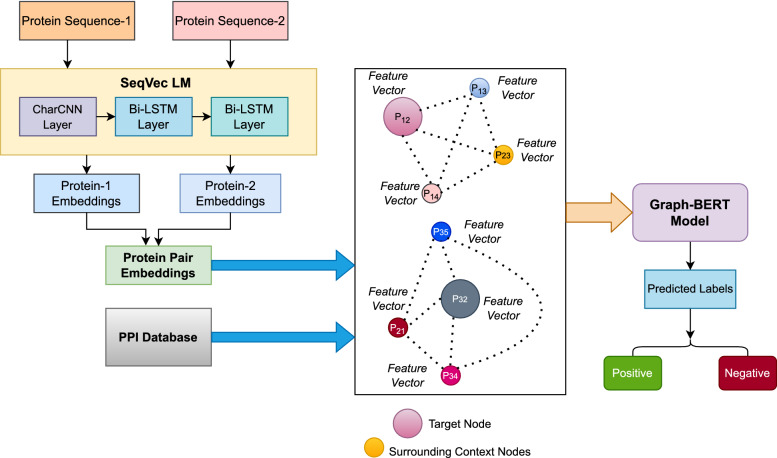
Illustration of the proposed approach.

### Protein sequence embedding

A protein’s amino acid sequence representation can be thought of as a language. The concept is to view each protein sequence as a sentence, with each amino acid acting as a word or character in the referred sentence. In order to model protein sequences, one can leverage the language model developed for NLP tasks. In this work, protein sequences are encoded into useful feature vectors using the *SeqVec*^22^ embedding technique. The *SeqVec* is context-dependent and is an adaptation of the bidirectional language model ELMO (Embeddings from language models)^44^. The configuration of the model is similar to that of the ELMO architecture with a few adjustments. Some of the changes made to the model include having a lesser number of tokens and making sure there are more unrolling steps to handle a longer protein sequence length. The number of tokens was reduced to 28. These are the following: 20 tokens for standard amino acids, 3 for ambiguous or unknown amino acids, 2 for uncommon amino acids, 2 for marking the beginning and ending of the sequence, and 1 for masking.

The *SeqVec* embedder consists of one character convolution (charCNN) layer^45^ followed by two bi-directional LSTM layers. Without taking into account the information from nearby words, the charCNN layer, which is the first layer, maps each amino acid of a protein sequence to a fixed-length (1024) latent space. The output of the charCNN layer serves as the input to the following layer, which is the first bi-LSTM layer. By concatenating 512 from the forward pass and 512 from the backward pass, each bi-LSTM layer has a dimension of 1024. A protein sequence of any length can be input to the ELMO-based *SeqVec* model, which is trained on UniRef50, and two types of embeddings are produced: *per-residue* (word-level) and *per-protein* (sentence-level). A protein sequence is first padded with <START> and <END> tokens to denote the beginning and end of the input before being passed to the first layer (charCNN) of the embedder. The pre-trained embedder creates an embedding of size (3, *L*, 1024) for an *L* length protein sequence by concatenating the outputs of three layers: one charCNN and two bi-LSTMs. Each amino acid in the protein sequence is associated with a 1024-dimensional embedding in each layer. The sum of the embeddings of the three layers yields the *per-residue* embedding. We obtain the *per-protein* embedding of size 1024 by averaging the *per-residue* embeddings across the *L* length of the sequence. As they generate uniform-length feature vectors for various-length protein sequences, we have used *per-protein* embeddings as feature vectors for nodes in the PPI network graph.

### PPI graph construction and classification

We have formulated the prediction of protein–protein interactions using the PPI network and sequence-based features as a node classification problem. First, we built the PPI network graph, where each node or vertex is a protein pair, and an edge is defined between two nodes if there is a common protein between them, as depicted in Figure 1. In this work, we have used the *SeqVec* language model to get a feature vector of size 2048 for each node, i.e., protein pairs. To learn low-dimensional features from a graph and then classify the PPI, we have used the Graph-BERT model. The prime advantage of this model is that it addresses both the suspended animation problem and the over-smoothing problem of other graph neural networks, such as GCN. The reason is that Graph-BERT does not rely on graph links for representation learning; instead, the model focuses on sampled linkless subgraphs. A suspended animation problem^17^ refers to a situation where the GCN model stops responding to training data and becomes unlearnable when the depth of the GCN model reaches a certain limit, known as the suspended animation limit. The GCN model is a variant of Laplacian smoothing that combines the features of a vertex with those of its close neighbors. The main reason GCNs are so effective is the smoothing process, which makes the features of vertices in the same cluster similar and significantly simplifies the classification task. The output features, however, may be over-smoothed^18^ if a GCN is deep with multiple convolutional layers, making it difficult to distinguish between vertices from various clusters. The working steps of the Graph-BERT-based approach are as follows:

- The input to the Graph-BERT model is the PPI network graph with node features.
- Graph-BERT is trained using linkless subgraph batches sampled from the input graph rather than the entire graph, $G_{ppi}$. So, the next step is to perform the batching of linkless subgraphs. To control sample-related randomness, the Graph-BERT model uses the *top-k intimacy* sampling strategy for sampling the subgraphs from the input graph. This sampling method is based on the graph intimacy matrix $S \in R^{\vert V \vert \times \vert V \vert }$, where entry *S*(*i*, *j*) calculates the degree of intimacy between nodes, $v_i$ and $v_j$. The intimacy score *S* is based on the PageRank algorithm defined as: $S = \alpha \cdot (I-(1-\alpha )AD^{-1})^{-1}$. Here, $\alpha \in [0,1]$, $AD^{-1}$ is the column-normalized adjacency matrix with *A* as the adjacency matrix and *D* as the diagonal matrix of *A*. The intimacy matrix *S* defines the learning context for any target node $v \in V$ by covering both local neighbors of *v* and the nodes far away.
- The next step is to prepare the input node vector embeddings, which cover four parts: 1. Raw feature vector embedding is expressed as $e^x = Embed(x) \in R^{d_h \times 1}$, converts the raw feature vector (*x*) of each node *v* in subgraph $g_i$ into a shared feature space with $d_h$ as dimension. Here, *Embed* represents the fully connected layer for numerical input attributes. 2. Weisfeiler-Lehman (WL) absolute role embedding is expressed as: $e^r = Position\_Embed(WL(v))$. Here, $WL(v) \in N$ is the WL code for each node *v* based on the node’s structural roles in the graph data and is invariant for different sampled subgraphs as it is pre-computed using the entire graph. The term $Position\_Embed$ is borrowed from Vaswani et al.^20^ to preserve positional information. 3. Intimacy-based relative positional embedding is expressed as: $e^p = Position\_Embed(P(v)) \in R^{d_h \times 1}$. The term *P*(*v*) is a positional index of node *v* and is different for different sampled subgraphs for the identical node, *v*. By default, the *P*(*v*) is 0, and the nodes closer to *v* have a small positional index. 4. Hop-based relative distance embedding is expressed as: $e^d = Position\_Embed(H(v, v')) \in R^{d_h \times 1}$. The term $H(v, v')$ represents the relative distance of node *v* in subgraph $g_i$ in hops to $v'$ in the original input graph. The embedding $e^d$ is considered as the balance between the WL absolute role embedding and intimacy-based relative positional embedding.
- The initial feature vector for node *v* in subgraph $g_i$ is calculated as:
  2 $$\begin{aligned} h^0 = aggregate(e^x, e^r, e^p, e^d) \end{aligned}$$
  Here, the aggregate function is defined as the vector summation. This feature vector for all nodes in a subgraph $g_i$ is organized into a matrix, $(H^0)$, fed to the graph transformer-based encoder. This encoder updates the nodes’ feature vector recursively with several layers (*D* layers). The output of the $l_{th}$ layer is defined as :
  3 $$\begin{aligned} H^l = softmax\big (\frac{QK^{T}}{\sqrt{d_h}}\big )V + G\_R(H^{l-1}, X_i) \end{aligned}$$
  where $Q = H^{l-1}W^l_Q; K = H^{l-1}W^l_K; V = H^{l-1}W^l_V$. Here, $W^l_Q$, $W^l_K$, and $W^l_V$ are the weight matrices for query, key, and value, respectively. The term $G\_R(H^{l-1}, X_i)$ denotes the graph residual term introduced by Zhang et al.^17^. It enables each layer of the model to be fed with the nodes’ initial features $(X_i)$ or intermediate representations $(H^{l-1})$ to maintain the effective representations for the inputs. This term is added to overcome the suspended animation problem. $X_i$ represents the raw features for all nodes in the subgraph, $g_i$.
- The next step is the representation fusion, defined as $z_i = Fusion(H^D)$. The function *Fusion* is the average of representations of all nodes in the subgraph, $g_i$. $z_i$ is the final representation for the target node, $v_i$.
- The final step is to predict the label for each node in the graph. For that purpose, a functional component is attached to the Graph-BERT transformer module. The input to that component is the learned representation, $(z_i)$. The functional component is defined as $\hat{y_i} = softmax(FC(z_i))$.

### Experimental setup

In this work, we have used the Graph-BERT (https://github.com/jwzhanggy/Graph-Bert) model, which is trained on our PPI datasets using GeForce GTX 1080 as the computing infrastructure. The deep learning libraries we need to run the code are pytorch (https://anaconda.org/pytorch/pytorch), sklearn (https://anaconda.org/anaconda/scikit-learn), transformers (https://anaconda.org/conda-forge/transformers), and networkx (https://anaconda.org/anaconda/networkx). Based on the literature^19^, the values of the parameters are chosen as *attention head number: 2*, *hidden layer number: D=2*, *hidden dropout rate: 0.5*, *attention dropout rate: 0.3*, *subgraph size: k=7*, learning rate: 0.001. During model training, the *binary cross-entropy* loss is minimized with the help of *Adam* optimizer^46^. The maximum number of *epochs* is chosen as 200 with an early stopping method to reduce overfitting. The proposed architecture is trained using a standard 80:20 train-test split training approach. The predictive capability of the proposed model is measured in terms of the parameters including *accuracy*, *sensitivity*, *specificity*, *precision*, *F-score*, and *MCC*. We have also reported the average 5-fold cross-validation results with standard deviation (Std. dev) values for all PPI datasets.

**Table 2:** Test set results on a benchmark human PPI dataset of baselines and the proposed approach.

Model	Accuracy	Sensitivity	Specificity	Precision	F-score	MCC
*Modified S-VGAE*	97.90	98.90	93.82	98.09	98.50	93.56
Proposed approach (*ProtBert*)	96.18	97.12	95.24	95.28	96.19	92.37
Proposed approach (*SeqVec*)	99.10	97.92	100	100	98.94	98.19

## Results and analysis

Here, we analyze the results obtained by our method, which is followed by a comparison with previous studies and some designed baselines.

### Results on benchmark PPI dataset

The proposed graph-based approach is different from the existing graph-based method^15^ in three aspects: (1) Graph construction; (2) Node features; and (3) A graph-based neural network for learning low-dimensional features. The approach suggested by Yang et al.^15^ constructs a graph where each node represents a protein, whereas in our case, each node represents a protein pair. We have used a language model (auto-engineered) to generate the initial feature vector for each node, whereas previous work has used the conjoint-triad method (manually crafted). Last, we have used a transformer-based graph model designed to address the problems of existing graph neural networks, whereas the previous work has used graph convolutional neural networks, which may suffer from the problems of over-smoothing and suspended animation.

The test set results of the proposed approach are tabulated in Table 2 for the benchmark human PPI dataset in terms of several evaluation metrics. We have designed some baselines to validate the correctness and effectiveness of our approach. In baseline-1, also known as *Modified S-VGAE*, we have used the same model proposed by Yang et al.^15^ [S-VGAE (https://github.com/fangyangbit/S-VGAE)] but changed the feature vector of nodes with our features, i.e., obtained by the *SeqVec* method. Yang et al. have used the conjoint-triad (CT) method to get each node’s feature vector. Results are reported in Table 2 (*Modified S-VGAE*). The designed baseline’s *accuracy, sensitivity, specificity, and precision* are 97.90%, 98.90%, 93.82%, and 98.09%, respectively. To make fair comparisons, we downloaded the code provided by Yang et al. and carried out experiments on the human PPI dataset with different node feature vectors (Conjoint-triad-based and *SeqVec* language model-based). With the CT-based feature vector and S-VGAE model, we achieve an accuracy of 97.07% (less than that reported by Yang et al. in their paper), whereas an accuracy of 97.90% is achieved with language model-based features and the S-VGAE model. As it is evident, the performance of the S-VGAE using language model-based node features performs better than the CT-based features. The accuracy (99.10%) that we get by our method shows that the *SeqVec* language model-based features and Graph-BERT together yield better results than the earlier graph-based work^15^ to predict PPI. We have designed another baseline-2, which is similar to our proposed approach. The only difference is that we have generated embeddings for protein sequences in baseline-2 using the *ProtBert*^47^ language model. This language model is trained on the BFD-100 dataset and has employed the BERT model^21^ to generate the embeddings for protein sequences. The designed baseline’s *accuracy, sensitivity, specificity, F-score, and MCC* are 96.18%, 97.12%, 95.24%, 96.19%, and 92.37%, respectively, reported in Table 2 (Proposed Approach (*ProtBert*)). As evident, the Graph-BERT-based PPI model utilizing the *SeqVec* language model embeddings performs better than those using the *ProtBert* language model embeddings to categorize the protein–protein interactions.

### Results on other PPI datasets

We have also validated the proposed approach’s effectiveness on other PPI datasets, such as *C. elegan*, *Drosophila*, and *E. coli*. The test results on these datasets are presented in Table 3. The *accuracy* and *F-score* of *C. elegan*, *Drosophila*, and *E. coli* PPI datasets are {99.44%, 99.98%, 99.74%}, and {99.56%, 99.98%, 99.68%}, respectively. We have also presented the average of 5-fold cross-validation results of the suggested method in Table 4 to check if the proposed approach is able to generalize a pattern in results or not. The standard deviation values are presented inside parentheses. On training the proposed approach on the combined dataset (*C. elegan, Drosophila, and E. coli*), we achieved a test accuracy of 94.79%, a specificity of 94.77%, and a sensitivity of 94.81%. All the results mentioned here are statistically significant. The Welch’s *t-test*^48^ at 5% significance level has been conducted, which gives the *p* value. We get *p* values $<0.05$, which implies that the improvement in results is statistically significant.

**Table 3:** Test set results on other PPI datasets of the proposed approach.

Datasets	Accuracy	Sensitivity	Specificity	Precision	F-score	MCC
*C. elegan*	99.44	99.83	98.78	99.30	99.56	98.80
*Drosophila*	99.98	99.96	100	100	99.98	99.96
*E. coli*	99.74	99.62	99.82	99.75	99.68	99.46

**Table 4:** Average 5-fold cross-validation results with standard deviation values inside brackets on all PPI datasets of the proposed approach.

Datasets	Accuracy	Sensitivity	Specificity	Precision	F-score	MCC
Human	99.02 (0.13)	99.15 (0.95)	98.57 (1.19)	98.94 (0.88)	99.04 (0.10)	98.00 (0.28)
*C. elegan*	99.51 (0.05)	99.20 (0.59)	99.51 (0.6)	99.72 (0.33)	99.46 (0.13)	98.96 (0.11)
*Drosophila*	99.99 (0.007)	99.99 (0.01)	100 (0.00)	100 (0.00)	99.99 (0.007)	99.99 (0.01)
*E. coli*	99.78 (0.09)	99.63 (0.12)	99.89 (0.10)	99.85 (0.14)	99.74 (0.11)	99.55 (0.19)

### Comparison with existing techniques

To date, a lot of studies have been conducted to predict PPI employing artificial intelligence-based approaches. Following the state-of-the-art methods, we have divided the whole dataset into a training set (80%) to train the model and a test set (20%) to evaluate the model’s performance. Initial studies^40,42,49,50^ utilizing sequence-based feature vectors and traditional machine learning-based algorithms to learn relevant features have reported training accuracy ranging from 90 to 97.90% for the human PPI dataset. Our approach’s training accuracy is more than 99.5% with an average test accuracy of 99.02%, which indicates the superiority of our method over these traditional methods. We have also compared our work with recent studies employing the latest deep learning algorithms such as stacked auto-encoder^31^, CNN and LSTM^35^, graph-based approaches^12,15^, and multi-modal PPI models^38,39^. The results are summarized in Table 5. In Table 5, we have presented the test set results to make the comparisons between the suggested and previous studies, and the report illustrates the superior performance that our model delivers over the older one and validates the correctness of our proposed approach. We re-implemented the graph-based approach (S-VGAE)^15^ as the source codes and PPI datasets are publicly available, and we have used the same PPI datasets to demonstrate our PPI model’s performance. For the human PPIs dataset, the *accuracy* and *MCC* of the proposed approach are 2.09% and 6.86% greater than those of the earlier graph-based approach^15^. Table 6 reports the performance comparisons for other species in terms of *accuracy*. The tabulated results (Tables 5 and 6) prove the efficacy of the suggested method over the existing methods.

**Table 5:** Performance comparisons of our approach with earlier approaches for Human dataset.

Model	Accuracy	Sensitivity	Specificity	Precision	F-score	MCC
Sun et al.^31^	96.82	–	–	–	–	–
Li et al.^35^	98.36	97.68	–	98.89	98.23	96.72
Jha and Saha^38^	97.20	98.07	95.04	97.99	98.03	93.16
Yang et al.^15^	97.07	98.19	93.46	97.98	98.09	91.88
Jha and Saha^39^	97.52	98.20	95.92	98.26	98.23	94.07
Jha et al.^12^	98.13	**98.84**	96.18	98.62	98.73	95.20
Proposed approach	**99.10**	97.92	**100**	**100**	**98.94**	**98.19**

Best values are highlighted in bold.

**Table 6:** Performance comparisons of our approach with earlier approaches for other PPI datasets in term of accuracy.

**Species**	**Proposed**	Yang et al.^15^	Li et al.^35^	Sun et al.^31^	Guo et al.^25^
*E. coli*	**99.74**	95.61	95.94	93.23	95.28
*Drosophila*	**99.98**	96.71	98.38	93.48	96.23
*C. elegan*	**99.44**	94.69	98.66	97.86	97.32

Best values are highlighted in bold.

### Discussion

Current work to predict the interaction between proteins has highlighted two important aspects: (1) Language model-based feature extraction needs to be explored more as it captures important features directly from protein sequences. We have also shown that the *SeqVec* language-model-based features, with an accuracy of 97.90%, perform better than the conjoint-triad-based features, which have an accuracy of 97.07%. (2) The graph-based transformer model (Graph-BERT) outperforms its graph convolutional counterpart in learning low-dimensional node features.

In our previous work^12^, utilizing the molecular graph of proteins and residue-level features obtained by language models, we have shown that the graph attention-based PPI model using *SeqVec*-based residue embeddings performs better than those of *ProtBERT*-based embeddings. Current work has also shown the same pattern of results. The proposed approach based on *SeqVec*-based protein embeddings (accuracy: 99.10%) outperforms the PPI model based on *ProtBERT*-based protein embeddings (accuracy: 96.18%). In the future, we will explore other language models to generate embeddings for protein sequences and analyze the results.

To further analyze the predictive capability of the proposed approach, we trained our model on imbalanced datasets. We created the imbalanced datasets by randomly selecting interacting and non-interacting pairs in different ratios. The test results on the human PPI dataset, along with the chosen ratio (interacting:non-interacting), are presented in Table 7. From these results, we can infer that the proposed approach performs well on imbalanced datasets as well. We also created imbalanced datasets with interacting and non-interacting samples in a 1:10 ratio to assess the proposed approach’s performance on skewed datasets of other species. The test set results are presented in Table 8. Based on the sensitivity and specificity values reported in Tables 7 and 8, we can conclude that our approach performs well on skewed datasets.

**Table 7:** Proposed approach’s test results for different ratios on human PPI dataset.

Ratio	Accuracy	Sensitivity	Specificity	Precision	F-score	MCC
1:2.5	98.53	99.89	97.92	95.58	97.69	96.67
1:5	98.63	97.42	98.89	94.78	96.08	95.27
1:10	99.44	99.06	99.47	94.62	96.79	96.51

**Table 8:** Proposed approach’s test results for other PPI datasets with interacting:non-interacting ratio as 1:10.

Species	Accuracy	Sensitivity	Specificity	Precision	F-score	MCC
*C. elegan*	99.18	99.99	99.11	90.62	95.08	94.77
*Drosophila*	99.96	99.66	99.99	99.99	99.83	99.82
*E. coli*	99.66	98.75	99.75	97.53	98.13	97.95

## Conclusion

In this work, we devise the PPI prediction problem as a node classification problem. Firstly, we build the protein network graph using the PPI database and assign each node (protein pair) a feature vector. The feature vectors are generated using the *SeqVec* language model from protein sequences. The Graph-BERT is used to learn the low-dimensional embeddings for graph nodes, which are input to the fully connected layer (FC). The output of FC is passed through the softmax layer to predict the PPI labels. The obtained results showcase the superiority of the suggested approach over previous work, including the graph-based approach by Yang et al. (S-VGAE)^15^. The final improvements can be summarized under the following pointers: (1) On replacing the conjoint-triad-based feature vector of the existing work (S-VGAE) with *SeqVec* language model-based embeddings (modified S-VGAE), we could find the accuracy improving from 97.07 to 97.90%. ( 2) By reformulating our problem as a node-classification problem and then using Graph-BERT with the language model-based embeddings, we could find the accuracy improving from 97.90 to 99.10%. Based on these experimental results, we can infer that the use of the pre-trained language model and Graph-BERT together boosts the predictive capability of the PPI model. Future work aims to explore other pre-trained language models to generate embeddings for protein sequences. Moreover, we will explore the use of other sources of protein information, such as gene co-expression, which can be utilized as a node feature vector in a PPI network graph.

## Data Availability

The source code for training and data to train the model are available at https://github.com/JhaKanchan15/PPI_GBERT. The dataset used in this study is available at https://github.com/fangyangbit/S-VGAE/tree/master/data.
